# Factors contributing to the low number of blood donors among employed residents in Oshatumba village, Namibia

**DOI:** 10.4102/phcfm.v15i1.3680

**Published:** 2023-04-24

**Authors:** Daniel O. Ashipala, Medusalem H. Joel

**Affiliations:** 1 Department of General Nursing Science, Faculty of Health Sciences and Veterinary Medicine, School of Nursing and Public Health, University of Namibia, Rundu, Namibia

**Keywords:** blood donation, blood donors, voluntary, education, uptake

## Abstract

**Background:**

Blood transfusion plays a significant role in maternal and child-care interventions, as well as by saving lives following natural disasters. Ignorance and fear among the general population in Namibia limit the numbers of blood donors, leaving the Namibian Blood Transfusion Services (NAMBTS) with insufficient donations for hospital patients. A review of the literature did not disclose publications on the factors that contribute to the low number of blood donors in Namibia, despite the urgent need for an increased pool of blood donors.

**Aim:**

The aim was to explore and describe the factors contributing to the low number of blood donors among the employed residents of the Oshatumba village, Oshana Region, Namibia.

**Settings:**

Interviews were conducted at a peri-urban village located in the eastern part of the Oshakati District in the Oshana Region.

**Methods:**

A qualitative methodology utilising explorative, descriptive and contextual strategies. Data were collected by means of individual, in-depth, semi-structured interviews with 15 participants, who were selected through convenience sampling.

**Results:**

The study discovered three themes: (1) the concept of blood donation; (2) factors contributing to low blood donations and (3) practical suggestions to increase the low uptake of blood donations.

**Conclusion:**

The findings of this study revealed that individual health status, religious beliefs and misconceptions associated with blood donations are among the factors that cause a low level of blood donations.

**Contribution:**

The research findings can be used to develop strategies and targeted interventions to increase the number of blood donors.

## Introduction

Blood is integral to human life and has no substitutes,^1^ which is why blood donors are critical to ensuring that life-saving blood transfusions can take place to support all manner of medical procedures.^2^ Indeed, transfusions are a basic emergency intervention in healthcare facilities around the world and play a considerable role in reducing mortality and morbidity. In addition, blood transfusion plays a significant role in maternal and child-care interventions, as well as by saving lives following natural disasters. Despite this, in many countries, there is an inadequate supply of blood available to meet the demand,^3^ which is rising in most countries because of an increase in life expectancy, as well as accidents, diseases such as anaemia and cancer and pregnancy-related complications.^4^ According to Abderrahman and Saleh,^5^ in excess of a million units of blood are donated every year in Jordan, yet millions more are needed to meet the growing demand. A concerted effort by governments is thus needed to not only identify the motivational factors that affect blood donation but also to recruit low-risk donors, particularly in the developing world.^5^

More than 70 countries had a blood donation rate under 1% in 2020,^6^ with the donation rate in developed countries being 33.1 per 1,000 people compared with only 4.6 donations per 1,000 people in developing countries. In excess of 112 million blood donations are collected every year, with approximately half coming from developed countries, yet those countries make up just 19% of the world’s population.^2^

Blood donations from voluntary non-remunerated donors (VNRD) were reported to have increased by 10.7 million donations from 2008 to 2013.^6^ In the Ivory Coast, a Sub-Saharan African country, the blood transfusion system comprises three blood transfusion centres, 11 blood transfusion satellite centres and five blood collection sites, all of which are under the supervision of the National Blood Transfusion Service (NBTS). Although their activity reports show an increase in blood donations every year, their sole reliance on volunteer non-remunerated donors does not address the inadequacy of blood supply in the country.^7^

In Malawian, hospital blood banks often ran dry and communities struggled to access safe and adequate blood.^8^ In 2004, given this critical shortage of safe blood products, the government founded the Malawi Blood Transfusion Service in line with World Health Organization (WHO) guidelines, which aimed to ensure the storage of blood products under strict measures of quality control.^9^ Similarly, between 2014 and 2016, Nigeria, Lesotho, Gambia and Ghana saw a decline in blood supply, yet Ethiopia had a 40% increase in the number of VNRD.^9^

In Kenya, blood donations in the capital, Nairobi, have been declining; in 2006, 38 808 units of blood were donated compared with just 30 840 units in 2009.^10^ According to WHO,^6^ a country needs at least 1% of its total population to donate blood, but with a Ugandan population of 34 million, the country should ideally collect 340 000 units of blood. Uganda needs about 250 000 units of blood to be able to meet its national needs but could only collect 220 000 units of blood in 2014 because of a variety of challenges.^11^ The factors that affect voluntary blood donation range from socio-demographic to organisational, religious, weight, age, physiological and psychological factors.^12^ In Namibia between April and May 2020, the coronavirus disease (COVID-19) lockdown led to the Namibian Blood Transfusion Services (NAMBTS) experiencing a critical shortage of blood donations as most blood donor clinics were shut down.^13^ According to the NAMBTS, the country needs on average 2,800 units to be donated every month,^14^ yet just 0.8% of the population who are eligible to donate blood do so.^6^ The country thus faces a continual shortage of blood, which puts hundreds of lives in jeopardy.^13^ It was against this backdrop that the Namibia Qualifications Authority announced that they would partner with the NAMBTS on a blood donation drive in the hope of increasing the country’s blood supply.^15^ Although the target was set to collect at least 25 units, this was not met as only 14 people donated blood, six of whom were first-time donors.^15^

The WHO estimates that 3–5% of a country’s population should donate blood annually if its stock of blood and blood products is to remain at an acceptable level,^16^ yet despite considerable efforts and blood donation programmes being coordinated around the globe, there are still inadequate supplies of healthy blood and blood products – particularly in developing countries. Although the factors affecting voluntary blood donation are well documented globally, the factors that result in insufficient blood donations in Namibia are unknown. The Oshatumba village in Oshakati East is located near the Intermediate Hospital Oshakati, where the NABTS are located. This village has an estimated population of 505 employees who are well educated and eligible to donate blood, yet most of these employed residents do not do so. The low number of blood donations by eligible donors in the Oshatumba village suggests that there are factors that are being overlooked in the recruitment and retention of blood donors in the village. The reasons for this were unknown, however, as no study had been conducted on this village to explore the phenomenon. For this reason, this study aimed to explore which factors are contributing to the low volume of blood donations at the Oshatumba village, Oshana Region, Namibia. These findings will be used to provide information to the blood banks so that they can plan an effective strategy to increase and maintain a safe and adequate blood supply.

## Research aim

The aim of this study was to explore and describe the factors that contribute to the low number of blood donors in the Oshatumba village, in order to create strategies to increase blood donations.

## Research methods and design

The research applied a qualitative methodology utilising explorative, descriptive and contextual strategies. The study is wholly descriptive as it focused on identifying, examining and describing the experiences of non-blood donors in the Oshatumba village. The intention of qualitative research is to understand a phenomenon rather than explain or predict it,^17^ that is, qualitative descriptive designs aim to describe and document aspects of a situation as they naturally occur.^18^

### Population, sample and setting

**FIGURE 1:**
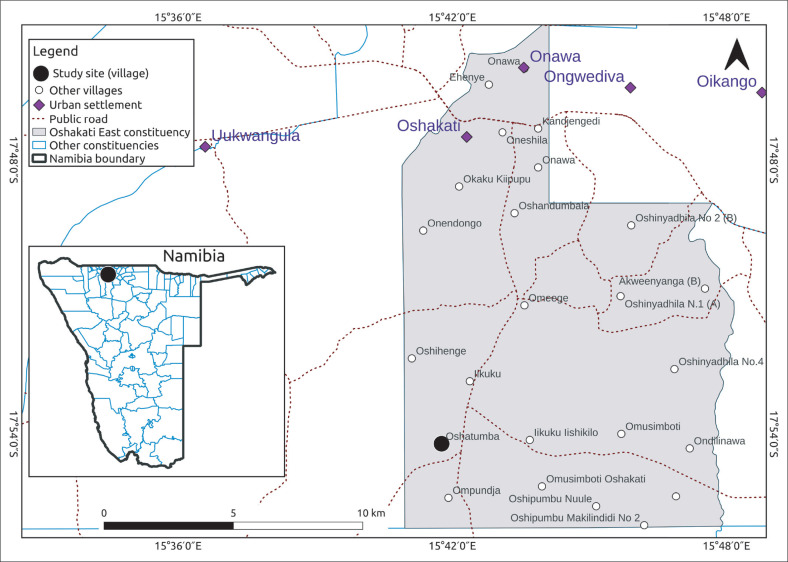
Oshatumba village map.

The research was conducted at the Oshatumba village, which is located in the eastern part of the Oshakati District of the Oshana Region in Namibia, with an approximate population of 6,500 residents (Figure 1). The public institutions that attract a productive age group to live in the village include, but are not limited to, schools (secondary, senior primary and primary), a health centre, agricultural offices, a Ministry of Gender Equality and Child welfare, a policy office and a regional council office, as well as many private institutions. These institutions served as settings where the data were collected.

The majority of the residents in this village are well educated, aged between 18 and 60 years and eligible to donate blood. In Namibia, mobile clinics are used to collect blood at numerous locations, from corporate headquarters to remote community centres, using contextualised recruitment to increase remote rural blood collection. Despite the proximity of the village to the Intermediate Hospital of Oshakati and its most educated residents, there has been a notably low volume of blood donors. In the Oshatumba village, most residents do not donate blood, yet the reason for this is unknown.

A sample is a subset of a study population that is used in statistics to represent the full group.^19^ The inclusive criteria in this study were: (1) be a resident of the Oshatumba village, (2) be between 18 and 60 years old, (3) be willing to participate and (4) be available at the time of study. For this study, 15 participants were conveniently selected from approximately 550 employees who were working in public and private institutions in the Oshatumba village. The participants were asked to describe the factors that contribute to the low volume of blood donations in the Oshatumba village, Oshana Region, Namibia, with a view to offering practical suggestions that could increase the number of donations. The researcher wrote to managers in the public and private sectors in Oshatumba village requesting that they distribute an invitation flyer to potential participants. This written invitation, which explained the purpose of the study, was placed on notice boards in tea rooms and board rooms at clinics, regional offices, head offices and schools, inviting interested participants to contact the researcher to participate in the study. Willing participants were invited to an information session about the purpose of the study and to provide answers to any questions they had. Interviews were conducted until data saturation was achieved, that is, it was felt that additional interviews would not yield any new information.^20^ Overall 15 subjects participated in the interviews, which took 4 weeks to complete.

### Data collection

Data were collected in August and September 2020, with one researcher conducting all the interviews under the supervision of an assigned study leader. The researcher secured written permission from the Oshana Regional Council, the constituency councillor for Oshakati East and the Village’s headman to conduct research in the Oshatumba village. The participants were required to provide written consent before they could participate in the research and were assured of their right to withdraw from the study at any time, although this was not encouraged. An interview guide was used to conduct the data collection; this was developed based on the research questions, the literature reviewed and the study’s objectives. The semi-structured interviews were held in line with the interview guide, which was created to capture why non-blood donors do not make blood donations. The date and time of the interviews were arranged to suit the schedules of the facility in which the interviews are to be conducted in order to minimise disruption to the services. All the interviews were conducted in English, despite the researcher offering a choice of languages. Each interview lasted between 50 min and 60 min. During the interviews, the researcher used an audio recorder while also taking notes, having first gained the participants’ permission to do so. Interviews were conducted until no new data emerged, which took place at the 15th interview. At this point, the data were said to be saturated. As the interviews were held in-person, all COVID-19 protocols were observed.

A pilot study was first conducted with four participants from the Othingo village, which is located north of Oshakati. This was performed in order to establish the relevance of the interview guide and questions, as well as their ease of use. The pilot participants were required to meet the same criteria as the population for the main study. No changes were made to the interview guide as a result of the pilot study. The following questions were posed:

- What is your understanding of the concept of blood donation?
- In your opinion, what factors contribute to the low number of blood donations in the Oshatumba village in the Oshana Region of Namibia?
- What mitigation strategies can be adopted to increase the number of blood donations in the Oshatumba village in the Oshana Region of Namibia?

### Data analysis

A qualitative content analysis method was used to manually conduct the data analysis,^21^ which was made up of the following six steps: (1) decontextualisation, (2) recontextualisation, (3) code category creation, (4) finalisation of rules for how codes are applied, (5) assignment of codes to the text and (6) drawing of conclusions and inferences linked specifically to the content data.^22^ During decontextualisation, the researchers familiarised themselves with the data by reading through transcripts to obtain a sense of the whole. Data were then broken down into smaller units of meaning. In the recontextualisation phase, the transcripts were read again alongside the final list of meaning units. At this stage, the researchers discarded unimportant information that did not match with the purpose and objectives of the study. In the categorisation step, themes and subthemes were identified, while in the compilation step, the researchers commenced with the writing up of findings. Finally, the analysed data were presented using the main research questions and objectives as the key themes.

### Measures to ensure trustworthiness

The trustworthiness of the study was ensured by using the criteria of Lincoln and Guba,^23^ namely credibility, dependability, transferability and confirmability. Credibility in this study was achieved through prolonged engagement with, and persistent observation of, the participants. The researcher remained in the field throughout the interview process in order to gain an in-depth understanding of the phenomenon. A tape recorder was used during the individual interviews, with those tapes being transcribed and the recordings and transcripts being compared with confirm that they were from the same participants. Transferability was achieved through a complete description of the research design and methodology used, as well as through the purposive selection of participants. The data collected were described as accurately as possible; full or thick descriptions of the experiences of the participants can be made available upon enquiry. Dependability was achieved through the use of audio recordings, and transcripts can be made available upon enquiry. The data collected are also supported with related literature, and the data collection methods are comprehensively described. In order to confirm the data, a consensus meeting with an independent coder, who was experienced in qualitative research and held a doctorate in nursing education, was utilised. In addition, verbatim transcription of the interviews and re-reading of these and the field notes enabled the researcher to get a better understanding of what the participants said about the factors that contribute to the low number of blood donations in the Oshatumba village.

### Ethical considerations

Approval for this study was granted by the University of Namibia’s School of Nursing Research Ethics and Review Committee (reference no. 11/2020) and the Ministry of Health’s Social Services Research Committee (ethical no. NS/2020). Informed consent was sought verbally from the participating respondents and through their signing of a consent form. Participation in the study was purely voluntary, and the interviewees were free to withdraw at any time, although this was not encouraged. The participants were also assured of anonymity, with the use of pseudonyms on the research tools instead of names, as well as confidentiality. The data were stored safely and will be disposed of according to the university’s policies.

## Findings

### Participants’ demographical data

The participants in this study were all residents of Oshatumba village, which is located in the eastern part of the Oshana Region (see Figure 1). Of the 15 participants, six were males and nine were females; nine were aged 18–30 years and six were aged 31–60 years and five were single and 10 were married. In terms of education, four had Masters degrees, four had undergraduate degrees, five had diplomas and two had only completed Grade 12. The participants were mainly civil servants who were employed by various ministries, such as teachers and police officers. They came from various religious denominations: five were Roman Catholic, five were Evangelical Lutherans, two were Anglican, two were Jehovah Witnesses and one stated ‘other’.

### Presentation of findings

The themes that emerged from the data analysis include: (1) the concept of blood donation, (2) factors that contribute to insufficient numbers of blood donors and (3) practical suggestions to increase the numbers of blood donors.

### Concept of blood donation

This theme describes the participants understanding of the concept blood donation. Participants demonstrated unique yet significant understanding on the concept blood donation. In addition, participants in this theme defined the concept blood donation as a voluntary procedure whereby a healthy person has blood drawn and the blood is used to save lives of those who need it:

‘It’s a voluntary process that is meant to save lives; when the healthy person donates blood to those who need it for survival.’ (P2, female, 25 years old)

‘Giving blood to those who need it most like to patients.’ (P9, female, 36 years old)

Blood is needed by a number of patients who experience life-threatening condition secondary to severe bleeding. As a result, the need for blood remains constant; as it helps patients survive heavy blood loss, for example, during surgical operations and traumatic injuries. Consequently, the participants reported that blood donation remain a lifesaving gesture:

‘It simply means that when a healthy person voluntary donates blood which will be given to those who need it. E.g. patients.’ (P15, male, 55 years old)

‘donating blood to those who need it most especially those involved in car accident or other diseases.’ (P8, female, 44 years old)

Participants also reported that blood is the most precious gift that anyone can give to another person through donating blood. Humans cannot live without blood because it is essential to life as it circulates through human body and delivers essential substances such as nutrients and oxygen to the body cells:

‘I understand it as that service to mankind that is geared toward the saving of people’s lives by donating blood and it’s a precious gift that can be given to someone.’ (P8, female, 44 years old)

### Factors contributing to the low uptake of blood donation

This theme is a description of the factors contributing to a decreased number of blood donors. Despite participants having displayed a good understanding of the concept of blood donation, they highlighted some various challenges contributing to the low uptake of blood donation. Cultural beliefs hamper blood donation as some participants reported that it is regarded as a taboo to donate blood in some cultures. In addition, blood donation is also coupled by the fear of blood being blood depleted should they donate regularly. Participants also reported the factors hampering blood donations, such as a lack of understanding about the procedure. In addition, some participants also indicated that they were scarred to donate blood as they believed they would be more susceptible to diseases such as human immunodeficiency virus (HIV) and acquired immunodeficiency syndrome (AIDS) during the donation process:

‘After donation of blood you can become vulnerable to infections.’ (P5, male, 18 years old)

‘If you donate your blood to anybody it might be used for other purposes.’ (P10, female, 30 years old)

‘… fear that it’s blood drawn from the body and their blood will get finished.’ (P6, male, 40 years old)

‘I want to donate but it is taboo in my culture so I am not really up for it.’ (P3, female, 55 years old)

‘… fear that it’s blood drawn from the body and their blood will get finished.’ (P11, male, 56 years)

Some participants also reported that their religious beliefs also contributed to the low uptake of blood donation, as they believe that humans must not sustain their life with another creature’s blood. These some beliefs bind some participants from donating blood:

‘Our Church principles bind us when it comes to the donation of blood and it is believed that if you receive a blood from a sinful person you too will become a sinner like that blood donor and when you die you will go straight to hell fire.’ (P9, female, 36 years old)

Participants reported that they have to travel long distances to go and donate blood as they stay far from blood donation sites. Findings from the study further confirmed that the lack of access to blood clinic is one of the factors contributing to the low uptake of blood donation. In addition, participants also reported that most of blood donors live in the remote rural area and had to travel long distances on foot to donate blood because of a lack transport money:

‘There distance to the donation site is very long and tiresome, as some people lack transport money to go to town to and donate blood.’ (P9, female, 36 years old)

### Practical suggestions to increase the number of blood donors

This is a theme description of participant’s suggestions on how to increase the number of blood donors. Participants expressed their views on how long distance contributes to the low uptake of blood donation. In order to hike the number of blood donors, participants suggested the provision of transport especially for those who live in remote areas to access the services:

‘Provision of transport to people who stay far from donation centers, especially those who live in remote areas, for them to be able to access services.’ (P5, male, 18 years old)

‘NAMBTS (must) come to people in order to cut down on the transport cost for donors.’ (P4, male, 33 years)

Their recommendations included that some of the staff members from the NAMBTS need to work on improving their professional conducts. Ethical conduct enhances fair treatment towards all individuals as they are treated with respect, creating a trusting relationship with clients. In addition, staff members need to address clients with courtesy and kindness to attract more clients to the blood transfusion services. Attitude of healthcare workers is a foundation as it makes a difference on how patients feel through respecting their rights and wishes. Participants suggested that the staffs at the donation sites need to improve their communication skills in a manner that is honest, respectful and treating clients with equality and compassion. Finally, participants indicated that healthcare workers need to value the diversity of life by creating a welcoming environment for donors:

‘Workers need to create good relationships with donors to make the environment conducive for the people to come and to continue donating blood.’ (P11, female, 31 years old)

‘Treatment of donor professionally will help.’ (P2, female, 25 years old)

‘NAMBTS Staff need to improve their customer service skills.’ (P20, male, 28 years)

In addition, participants highlighted that education plays a pivotal role in blood donors as it promotes knowledge, attitude and beliefs towards blood donation, which motivates blood donors among minorities. Creating awareness through the promotional campaign of blood donation increases the number of blood donors. Individuals lack the necessary information pertaining to blood donation as they do not understand the purpose of blood donation. Participants also suggested that all components of the media such as radio, TV, local English daily and social media such as Facebook can be effectively used to promote blood donation to the public. These teaching tools can be used to disseminate correct information and dispel myths associated with the issue of blood donation:

‘The mass media such as radio, TV, local English daily and social media such as Facebook can be used to disseminate correct information and dispel myths associated with regard to the issue of blood donation.’ (P1, female, 21 years old)

‘People need education on the importance of blood donation.’ (P3, female, 55 years old)

‘Dispelling myths conveying correct information can go a long way in increasing blood supply.’ (P7, female, 45 years old)

## Discussion

This study sought to explore the factors that contribute to the low number of blood donors in the Oshatumba village. This section presents a discussion of the findings in accordance with the themes that emerged from the study, namely the concept of blood donation, factors that contribute to the low number of blood donors and practical suggestions to increase the number of blood donors.

### The concept of blood donation

The participants described blood donation as being a voluntary process that is meant to save lives, that is, a healthy person donates blood to those who need it. This is in line with a study undertaken in Tanzania by Elias, Mauka, Philemon, Damian, Mahande and Msuya,^24^ who found that blood is the most donated tissue in medical practice and can be used as a tool in many life-saving medical treatments if used appropriately. Its transfusion from donors is an indispensable part of modern healthcare, which can save lives and improve health. The participants further described blood donation as a step of generosity towards serving humankind. However, blood donation is a social norm that needs not only personal motivations, but it is also influenced by institutional and sociocultural factors, which may dictate who is capable of donating blood and their reason for doing so.^25^

### Factors contributing to the low number of blood donors

The participants described a lack of information as being a major contributing factor for some people either refusing to or delaying donating blood. Al-Enizi, Al-Shammari, Al-Shammari and Al-Dhafiry^26^ similarly discovered that a lack of awareness, misconceptions, a lack of time, negative attitudes as well as fear of the blood donation process are to be blamed for the decrease in the number of blood donations. Some participants believed that after donating blood, a person becomes vulnerable to infections. So long as donors are healthy at the point of donation, as established by screening for anaemia and other signs of ill health, there is no evidence of risk of infections to donors. Although research has established a small risk of transfusion transmitted infections (< 5%), especially HIV, this risk is to the recipient of blood not to the donor, but which compromises blood safety.^27^

Participants in this study reported that an additional barrier to blood donation is distance to the blood donation site, as proximity plays a major role when it comes to motivating donors to return. A study conducted by Baig, Habib, Haji, Alsharief, Noor and Makki^28^ also found that an individual’s willingness to donate was influenced by the distance to the donation site. Participants in this study recommended the provision of transport to people who stay far away from donation centres, especially those who live in remote areas. Some participants highlighted that a lack of transport fees can also be a challenge. Similar findings were reported by Sharma, Madan, Venkatesh, Ichhpujani and Lal,^29^ where the majority of survey participants who did not routinely donate blood reported not having a convenient place to donate because of distance or a lack of transportation to the donation site.

Some participants in this study believed that blood donation is a sin, and everyone who donates blood will go to hell. Their beliefs prevent them from taking part in a blood transfusion, which is widely included in standard methods of life-saving treatments.^30^ This is similar to a study conducted by Wong, Mistovich and Morrison,^31^ who stated that receiving blood is considered to be the same as consuming blood by most Jehovah’s Witness members. Blood donations and transfusions are thus believed to be a reason for excommunication and a loss of eternal life.^32^ This is also in line with a study by Rajtar^33^, who explained that Jehovah’s Witnesses believe that the Bible prohibits Christians from accepting blood transfusions or donations. Participants in this study emphasised that community-based non-governmental organisations (NGOs) can be used to encourage potential donors to become actively involved in donor education, recruitment and retention programmes.

### Practical suggestions to increase the low number of blood donors

To bridge the identified gap between knowledge about blood donations and the act itself, the participants suggested a need for more community education regarding the importance of blood donations to dispel myths and address cultural and religious beliefs. Understanding the public’s perceptions and concerns, as described in this study, and establishing various platforms for dialogue and trust-building, are vital components to promoting blood donations.^34^ Participants in this study emphasised that community-based NGOs can be used to encourage potential donors to become actively involved in donor education, recruitment and retention programmes.

This includes placing greater emphasis on the fact that blood donations are not associated with health risks to donors who are healthy.^35^ Alreshidi and Sula^36^ suggested a need for future planning, with emphasis on educational programmes and awareness campaigns by organisations, as an effective approach to dispel misconceptions and motivate potential blood donors.

The participants in this study suggested that mass media such as radio, television (TV) and local news, as well as social media platforms such as Facebook, can be used to disseminate correct information to dispel myths associated with blood donation, which was also noticed by Zago, Silveira and Dumith.^34,35^ In addition, the study results suggest that transport to blood donation sites should be offered in order to boost blood donation. MacVinish, van Leeuwen and Hoetjes^37^ noticed that in the event of a lack of finance and/or transportation, organisations have to decentralise the service by utilising the knowledge of potential donors and community members to identify the necessary model to create access.

A proper community entry process, that, through community leaders as well as community liaisons, could facilitate more community involvement and thereby attract more blood donors. Blood donations could also be boosted through public awareness campaigns, for example by posters and annual events that honour donors.^38^

### Strengths, limitations and areas for further research

The strength of this study lies in the fact that the perceptions of participants regarding which factors contribute to the low number of blood donors in the Oshatumba village were considered from their emic perspectives. As the results of this study were obtained from one selected village, however, generalisation of the findings cannot be made to other villages. Further research could include a quantitative study that utilises a questionnaire in both urban and rural villages. This could also include a comparative section.

## Conclusion

The aim of the study was to explore and describe the factors contributing to the low number of blood donors among the employed residents of the Oshatumba village, Oshana Region, Namibia. This study identified several factors that limit blood donations, including religious beliefs, lack of information, lack of access to blood donation clinics, health concerns and unprofessional behaviours of blood donation staff. The findings of this study can be used to develop strategies and targeted interventions to increase the number of blood donations.
